# Osteochondral Lesions of Ankle and Knee. Will Future Treatments Really Be Represented by Custom-Made Metal Implants?

**DOI:** 10.3390/jcm11133817

**Published:** 2022-07-01

**Authors:** Massimiliano Mosca, Alberto Grassi, Silvio Caravelli

**Affiliations:** II Clinic of Orthopaedics and Traumatology, IRCCS Istituto Ortopedico Rizzoli, 40136 Bologna, Italy; massimiliano.mosca@ior.it (M.M.); alberto.grassi@ior.it (A.G.)

## 1. Introduction

Knee and ankle osteochondral lesions are structural defects of the cartilaginous surface and underlying subchondral bone which still represent a daily challenge for the orthopedic surgeon. Although etiology on a traumatic basis accounts for most cases, other causes are contemplated, including joint malalignments, instability, genetic predisposition, endocrine factors or avascular necrosis [1,2,3]. Optimal treatment is still the subject of debate.

Bone marrow stimulation procedures, osteochondral grafting and the osteochondral autograft transfer system (OATS) [4] are usually the first therapeutic step in young and active patients. However, these procedures are typically associated with donor site morbidity (pain, scar tissue and sensibility issues), incongruent grafts or graft resorption. Ferreira et al. [5] reported a complication rate of up to 41% after OATS surgery. Elderly patients with low functional requirements often benefit from a conventional joint replacement.

## 2. Discussion

However, there is a pool of patients who fall into the so-called “gap of treatment”—active patients suffering from osteochondral lesions in the context of an otherwise healthy joint. These subjects have often passed the age for biological treatment but are not yet eligible for early knee or ankle joint prosthesis or come to our observation after a failed biological intervention [6]. In the last two decades, the interest in this type of patient has increased, leading to the development and production of small metal prosthetic devices of “focal joint replacement” or “focal resurfacing”, with the aim of filling only the symptomatic cartilaginous lesion of the talar dome or femoral condyles. After the initial enthusiasm for good clinical and functional results, described in the literature in different studies [2,7,8,9], the complication rate reduced the expectations of these implants. The technical difficulties of the implant, the malpositioning and the particular and peculiar joint geometries of the ankle and knee can strongly influence the surgical result. Despite the design being developed to adapt to the joint surfaces, minimal changes in the implant positioning could create problems during walking and not be tolerated in biomechanically complex and congruent joints [2]. In addition, high rates of re-intervention have been described, whether or not related to the implant itself. In particular, repositioning or removal of the prosthesis, subchondral periprosthetic radiolucency, joint space narrowing and cyst formations around the implant screw have been reported [2,10].

In this panorama, new custom-made talar and condylar devices (Episealer^®^) have recently been designed and developed to address these technical issues. These CT-based patient-specific mini-metal prostheses are produced following the patient’s joint anatomy, location and volumetric characteristics of the osteochondral lesion. They aim to represent the next step in advanced resurfacing techniques, improving clinical outcomes and avoiding the specific disadvantages of standard metal resurfacing. Despite being newly designed implants, good short-term results have already been published, reporting a failure rate of 2.5% [11,12]. A study by Moewis P et al. [6] evaluated these new implants at a 12-month follow-up, showing that after the condylar implantation, the knee kinematics were physiological with a medial pivot, lateral femoral rollback and coupled axial pattern, and external rotation during flexion.

This short editorial aims to ask questions and propose new long-term research approaches about the possibilities of custom-made metal implants, which are already revolutionizing the concept of total prosthetics, to improve clinical and radiological outcomes in patients suffering from primary or secondary osteochondral lesions following the failure of previous biological treatments. Moreover, we will focus on the duration and possible different complications, with respect to the biomechanics of large joints, pitfalls and technical tricks, and the cost/benefit ratio for the patient and the health protection entities.
